# Adaptive photoperiod interpretation modulates phenological timing in Atlantic salmon

**DOI:** 10.1038/s41598-023-27583-7

**Published:** 2023-02-14

**Authors:** Tina Oldham, Frode Oppedal, Per Gunnar Fjelldal, Tom Johnny Hansen

**Affiliations:** Institute of Marine Research (IMR), Matre Aquaculture Research Station, Matredal, Norway

**Keywords:** Phenology, Marine biology

## Abstract

Photoperiod, the portion of 24-h cycle during which an organism is exposed to illumination, is an important phenological cue in many animals. However, despite its influence on critical biological processes, there remain many unknowns regarding how variations in light intensity translate into perceived photoperiod. This experiment examined how light intensity variations affect perceived photoperiod in Atlantic salmon (*Salmo salar*) to determine whether photoperiod interpretation is, a) fixed such that anything above a minimum detection threshold is regarded as ‘illumination’, or b) adaptive and varies with recent light exposure. To do this we compared the frequency of smoltification and sexual maturation between groups of male parr which were exposed to one of eight light regimes on a 12:12 cycling regime (12-hour day/12-hour night). The eight regimes were divided into two treatments, four with ‘High’ daytime light intensity and four with ‘Low’ daytime light intensity. The ‘High' and ‘Low' intensity treatments were each sub-divided into four groups for which the subjective ‘night’ light intensity was 100%, 10%, 1% and 0% of the daytime light intensity, with four replicate tanks of each treatment. The results show that above a minimum detection threshold, Atlantic salmon have adaptive photoperiod interpretation which varies with recent light exposure, and that adaptive photoperiod interpretation modulates the timing of the parr-smolt transformation and sexual maturation. Further, we show that photoperiod interpretation varies between closely related families. Given the influence of phenological timing on species survival, our results reveal a critical role for integration of photoperiod interpretation in attempts to understand how geographically shifting thermal niches due to climate change will affect future populations.

## Introduction

The daily cycle of light and dark plays a critical role in the timing of development, migration and reproduction in many species^1–4^. Though conceptually simple, practically defining daylength is difficult. Which conditions each animal recognizes as ‘illumination’ differs with anatomy, habitat and physiology. While one species may have highly sensitive photoreceptors and interpret the dim light of high latitude mid-winter as ‘illumination’, a less light sensitive species may not detect such dim light at all and recognize the same conditions as darkness^5^. Further, just because an individual is able to physically detect light does not necessarily mean that they will interpret such conditions as ‘illumination’^5–8^. It has long been understood that intensity of illumination alters how it is perceived^9^.

Based on studies examining melatonin fluctuation in response to varied light exposure regimes, some vertebrates appear to have a fixed illumination threshold above which all conditions are interpreted as day, whereas others have an adaptive illumination threshold which changes with recent photic history^5–8,10,11^. Among teleosts, dramatic differences in photoreception and photoperiod perception have been detected. European sea bass are at least 10 times more sensitive to light than Atlantic salmon, while Atlantic cod are 100 times more sensitive yet again^5,11^. In addition, in an ex vivo trial where pineal organs were removed from cod, increased day-time light intensity exposure significantly reduced night-time light sensitivity and shifted melatonin response curves^5^. Importantly though, these findings are based entirely on the response of isolated pineal organs to different light exposure regimes, and only examine melatonin response. In isolation the pineal has different sensitivities to that of the whole animal^11^, and the role of melatonin in the control and timing of phenology in teleosts remains unclear^3,12^.

Salmonids are an ideal group in which to study photoperiod interpretation because they exhibit no apparent diel rhythm when held in constant conditions, unlike other vertebrates^11,13–15^. As a result, salmon experience no conflicting signals of internal origin to those of the environment, allowing for the direct examination of how environmental exposure affects phenological timing. For example, by adjusting temperature and duration of light exposure the timing of both the parr smolt transformation and sexual maturation can be quickly and easily be modified in Atlantic salmon^16^.

With this in mind, we used parr from four all male Atlantic salmon (*Salmo salar*) families in a common garden setting to study the phenological implications of differential photoperiod interpretation. Four replicate tanks of each group were exposed to one of eight light regimes on a 12:12 cycle. The eight regimes were divided into two treatments, four with high daytime light intensity (High) equivalent to winter mid-day (12:00) at 60°N, and four with low daytime light intensity (Low) similar to that of sunrise. The High and Low intensity treatments were each further sub-divided into four groups for which the subjective “night” light intensity was 100% (continuous light, unchanging; LL), 10%, 1% and 0% (true 12:12 cycle with complete darkness; LD) of the day light intensity. Based on estimations in previous studies of a lower detection limit of salmon between 0.05 and 0.07 µmol m^−2^ s^−1^^11,17^, intermediate light intensities of 10% and 1% were chosen so that the “night” light intensity in the High treatment group would be above the expected detection threshold, while in the Low intensity group it would be near or below threshold, respectively. By comparing the frequencies of smoltification and sexual maturation between treatments, we show that, (1) above a minimum detection threshold, Atlantic salmon have adaptive photoperiod perception which changes with recent photic history, (2) adaptive photoperiod interpretation modulates the timing of both the parr smolt transformation and sexual maturation, and (3) photoperiod interpretation varies among closely related populations.

## Materials and methods

### Animals

Four all male hatchery reared Atlantic salmon families were sourced from the Matre Research Station at the Institute of Marine Research, Norway. The four lines were derived from crosses between two Aquagen background females and two YY males^18^.

### Husbandry

On 16 January 2019, eight days prior to the start of the experiment, all 1768 parr (103 ± 24 g, mean ± SD) were anaesthetized, intra-peritoneally tagged with 2 × 12.1 mm FDX RFID tags (RFID solutions, Norway) and transferred to experimental tanks. Each of the four families were divided evenly amongst 32 grey 1 m × 1 m tanks. Throughout the trial, tanks were supplied with flow through freshwater at a rate of 20 L min^−1^, and fish were fed standard commercial feed (Skretting, Norway) in excess between 08:00 until 18:00. To maximize the likelihood of sexual maturation we aimed to run the experiment at a relatively high temperature of 16 °C based on previous work^16^. From 17 January, temperature was increased in all tanks by approximately 2 °C day^−1^ until the experimental temperature of 15.9 ± 0.2 °C (mean ± SD) was reached. Dissolved oxygen was continuously monitored and did not fall below 85% saturation.

### Light treatments

Each tank was equipped with two Phillips TL-D LIFEMAX Super 80 18 W fluorescent tubes which were replaced at the start of the trial. For at least six weeks prior to the start of the experiment, all families were exposed to a simulated natural photoperiod. On 24 January 2019, the photoperiod was adjusted to one of eight treatments on a 12 h (day; 08:01–20:00): 12 h (night; 20:01–08:00) cycle. There were four high light intensity groups (High) in which the light intensity was 70.4 ± 2.2 µmol m^−2^ s^−1^ during the day, and four low light intensity groups (Low) in which the light intensity was 0.99 ± 0.10 µmol m^−2^ s^−1^ (mean ± SD) during the day. The High and Low treatments were each further subdivided into four groups for which the subjective night light intensities were 100% (continuous light, LL), 10%, 1% and 0% (darkness, 12L:12D, LD) of the corresponding day light intensity (Table 1). All lights were controlled by a custom system with automatic timer and power adjustment. Light treatments were created using a combination of shade cloth and light source output adjustment. Each light exposure group was replicated in four tanks (32 tanks total) with the families distributed amongst tanks in a common garden design. Light intensity was measured using a LI-1500 logger equipped with an LI-193 spherical quantum underwater radiation sensor (LI-COR Biosciences, USA) just below the waters’ surface in the middle of each tank with the lid closed. Overhead lights were turned off for the duration of the experiment, and all work in the experimental rooms was performed using a red LED torch. The final sampling was performed on 10–11 April 2019, after 11 weeks of exposure to the experimental light regimes.

**Table 1:** Light exposure treatments.

Group ID	Light Intensity	Day	Night	Night/Day
		(08:01–20:00)	(20:01—08:00)	(%)
High LL	High	73.5 ± 2.0	73.5 ± 2.0	100%
High 10	High	69.3 ± 0.96	6.8 ± 0.5	10%
High 1	High	69.5 ± 1.0	0.6 ± 0.10	1%
High LD	High	69.3 ± 0.96	0	0%
Low LL	Low	1.0 ± 0.08	1.0 ± 0.08	100%
Low 10	Low	1.0 ± 0.05	0.1 ± 0.0	10%
Low 1	Low	1.0 ± 0.03	0.01 ± 0.0	1%

*Units = µmol m^−2^ s^−1^.

Recorded light intensity (µmol m^−2^ s^−1^) measurements of each group (mean ± SD).

### Seawater challenge

On 13 February feeding was stopped to all tanks. Twentyfour hours later, after 22 days of exposure to the experimental light treatments, 16 fish from each treatment (4 tank^−1^) were sampled as freshwater controls and 32 fish from each treatment (8 tank^−1^) were transferred into eight identical 1 m × 1 m tanks filled with 16 °C seawater at 34.5 ؉ in a randomized block design. After 24 h exposure to seawater, fish were euthanized and samples collected.

### Sampling protocol

Prior to sampling all fish were euthanized via immersion in 0.5 g L^−1^ Finquel MS-222 in buffered freshwater, or seawater after the seawater challenge test. After a blood sample was drawn from the caudal vein using a heparinized syringe, each fish was scanned for the PIT tag, measured and weighed. Blood samples were briefly stored ice until they were centrifuged for 1.5 min at 13,200 rpm at 4 °C. Plasma was then extracted and briefly stored on dry-ice until transfer to -80 °C. After blood sampling the fish were exsanguinated, the adipose fin was removed and stored in ethanol, the fish were sexed, and the gonads removed and weighed.

### Sample processing

The concentration of plasma cortisol was measured with an ELISA assay kit (IBL International GmbH), and plasma osmolality was measured by freeze point determination with a Fiske 210 Micro-Sample Osmometer (Advanced Instruments). Other plasma parameters, including pH and the concentrations of Cl^−^, Na^+^, Ca^++^ and K^+^, were measured with an ABL90 FLEX blood gas analyser (Radiometer).

### Data analyses

Condition factor (K) was calculated as K = 100 × body weight (g)/fork length^3^ (cm)^19^. Specific growth rate (SGR) was calculated as: SGR = (e^G^−1) × 100, where G = (ln (W_2_)−ln (W_1_))/(t_2_−t_1_),W_2_ and W_1_ are body weight at times t_2_ and t_1,_ respectively. The gonadosomatic index (GSI) was calculated as GSI = (gonad weight × 100)/body weight. To examine the effect of light exposure treatment on GSI, the natural log of the response ratio was calculated as: lnRR = ln(µT/µC), where µT is the treatment group response and µC is the control (0%) group response. A histogram of GSI at the final sampling displayed a bimodal distribution with a large peak centred on 0.04 and a second, smaller peak centred on 6.6. Based on previous empirical studies immature salmon have a GSI of less than 0.11^18,20^, so all fish in the initial peak with GSI < 0.11 were classified as immature, while the remaining fish were classified as maturing. Single factor anova were used to test for differences in size between groups at the start of the trial. A significance level of α = 0.05 was used throughout.

To evaluate the impact of light exposure treatment on smoltification, generalized linear mixed models (GLMM) were created with the package ‘glmmTMB’^21^ in R (R Core Team 2018). SGR, K and each of the plasma parameters (osmolality, pH, [cortisol], [lactate], [Cl^−^], [Ca^++^], [Na^+^] and [K^+^]) were modelled as a function of the interaction between *day light intensity* (factor: High/Low) and *night light %* (factor: 0, 1, 10, 100)*.* To account for the dependency among individuals from the same tanks, a mixed model was applied with *tank* as random intercept. SGR and K were modelled using a gaussian distribution with an ‘identity’ link function, whereas plasma parameters were modelled using a gamma distribution and ‘log’ link function to ensure no negative values were fitted. Model assumptions of independence, homogeneity and normality were examined by looking for patterns in the plots of Pearson residuals versus fitted values and versus each covariate. Pair wise comparisons of the light treatment groups were performed by computing and contrasting estimated marginal means (least square means) with Tukey-adjusted *P-*value correction for multiple comparisons using the ‘emmeans’ package^22^.

To evaluate the impact of light exposure treatment on maturation, a binomial GLMM was fitted with the package ‘lme4’^23^ in R (R Core Team 2018). The full model of probability of maturation included *light treatment* (factor–8 levels: High/Low, 0, 1, 10, 100%), *family* (factor: A–D) and *initial weight* (continuous), with *tank* included as random intercept. Model selection was performed using the drop1 function and Akaike information criterion (AIC). All covariates were influential and remained in the final model. Model validation was performed by creating a binned plot where the average expected values from the logistic regression were compared to the average residual values within each ‘bin’.

### Ethical statement

Experimental protocols were approved by the animal experimentation administration (Forsøksdyrforvaltningen—FDF) in the Norwegian Food Safety Authority (NFSA), permit number 17665. All welfare and use of animals were performed in accordance with the Norwegian Animal Welfare Act of 19th June 2009, enforced on the 1st of January 2010. In addition, all personnel involved in the data collection underwent training approved by the NFSA, which is mandatory for all personnel handling fish. This study is reported in accordance with the ARRIVE guidelines.

## Results

This study examined photoperiod perception in Atlantic salmon by comparing the frequency of smoltification and sexual maturation between groups of parr exposed to light regimes of varying intensities. Salmon displayed a fixed in vivo minimum light detection threshold between 0.01 and 0.1 µmol m^−2^ s^−1^, above which photoperiod interpretation was adaptive.

### Frequency of sexual maturation

**Figure 1:**
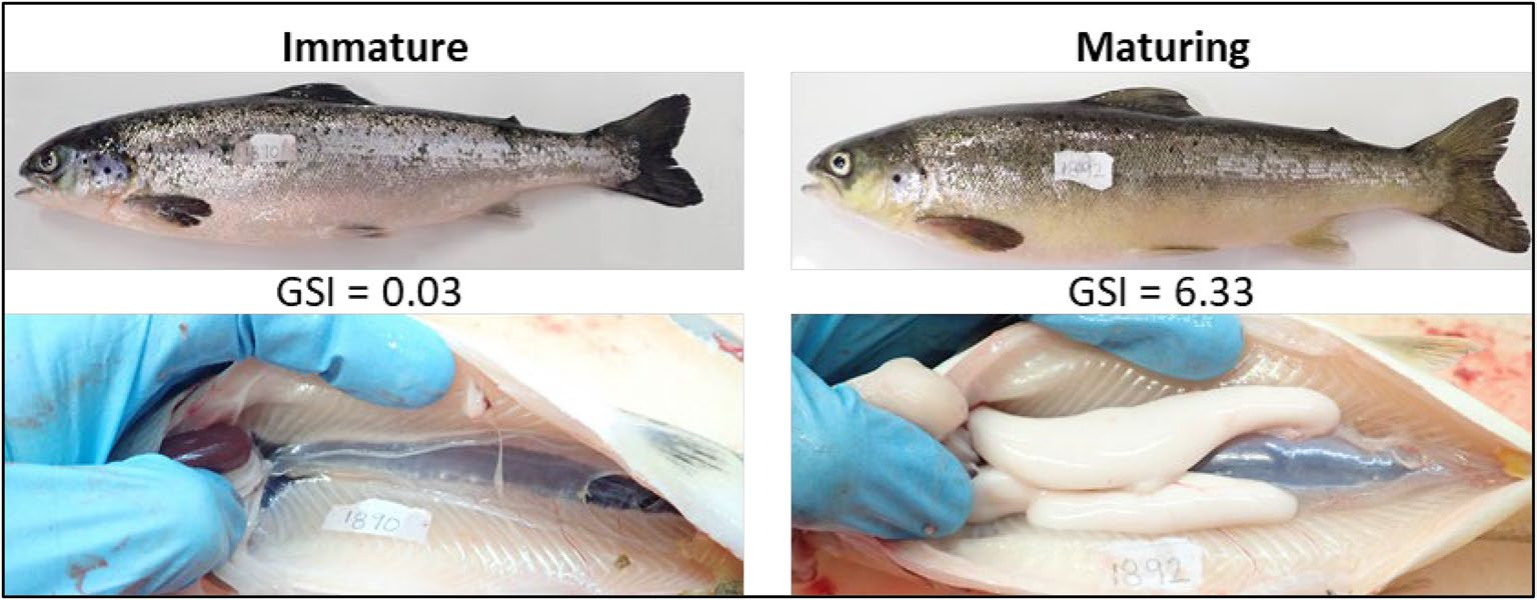
Typical examples of immature and maturing Atlantic salmon. Photographs showing the external appearance and gonads of fish classified as either immature (GSI < 0.8) or maturing. Photographs by Tina Oldham.

**Figure 2:**
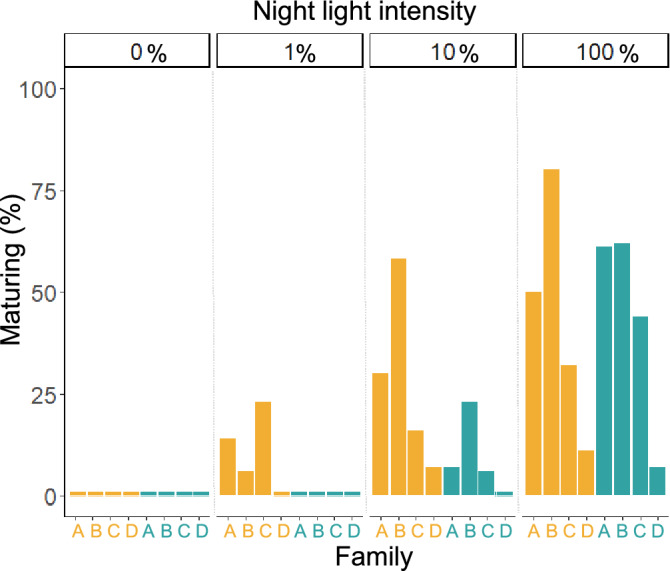
Impact of light treatment and family on maturation status. Percent of individuals maturing in each light treatment and family. High light intensity groups are presented in orange, while the low light intensity groups are presented in green.

During the first sampling, after 22 days of exposure to experimental light regimes, GSI ranged from 0.01 to 0.349, with a total of 20 of the 365 sampled individuals maturing. On average the immature fish weighed 100 ± 18 g, while maturing fish were larger averaging 118 ± 15 g. The maturing fish were evenly divided between the High and Low intensity treatments. At the final sampling, GSI ranged from 0.01 to 10.4, and after 77 days of exposure to the experimental light regimes, a total of 185 fish were maturing (Fig. 1). No fish in either negative control group (LD, 0) matured when held in 12 h light and 12 h complete dark, while an average of 33% and 36% of fish were maturing in the High LL and Low LL groups, respectively (Fig. 2).

### Minimum detection threshold

**Figure 3:**
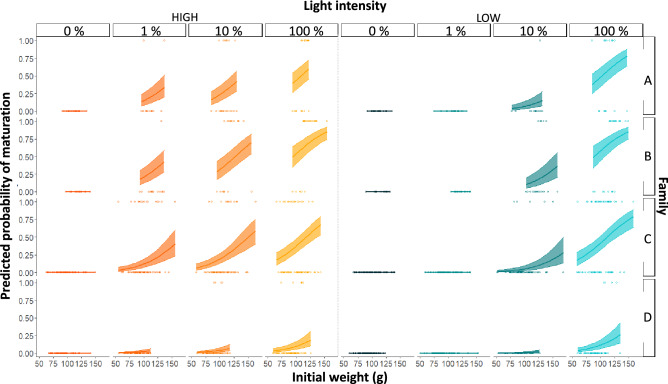
Impact of light treatment, family and initial weight on probability of maturation. Solid lines and shaded areas display the fitted values ± 95% confidence intervals from the generalized linear mixed model. High light intensity groups are presented in orange, while the low light intensity groups are presented in green. Individual data points are presented as open circles.

**Figure 4:**
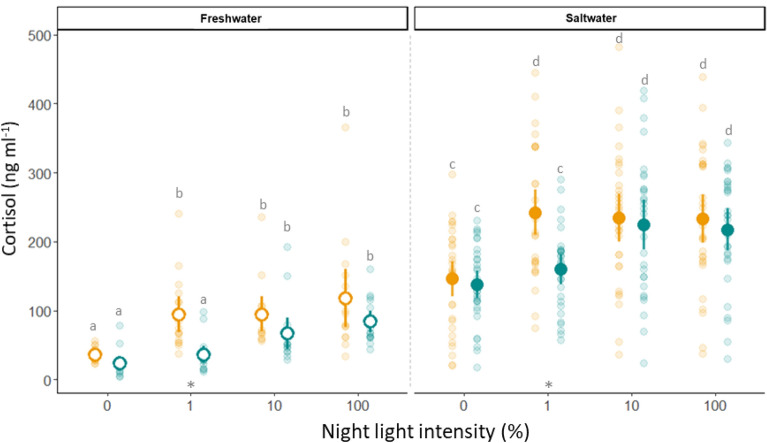
Impact of light treatment and saltwater challenge on cortisol. Plasma cortisol concentrations after 22 days of exposure to light treatments. Open points display the means ± 95% confidence intervals of GLMMs from control samples collected in freshwater, while solid points display the means ± 95% confidence intervals after 24 h acute exposure to 34.5 ppt saltwater. Faded points present individual measurements. High light intensity groups are presented in orange, while the low light intensity groups are presented in green. Significant differences between treatments within salinity groups are indicated by different lower-case letters.

**Figure 5:**
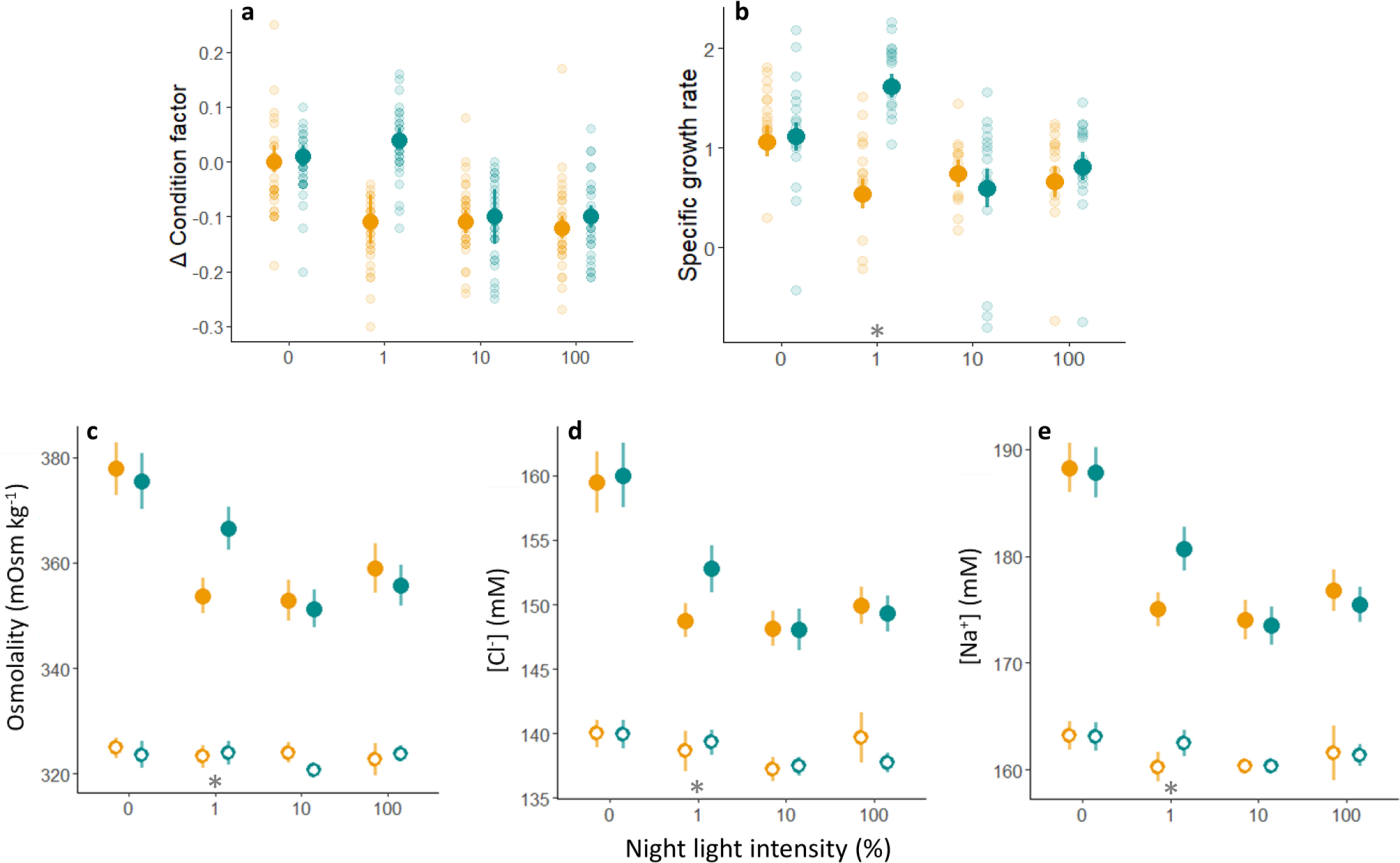
Impact of light treatment on smoltification. Fish status (**a**: Δ condition factor, **b**: specific growth rate) and plasma parameters (**c**: osmolality, **d**: [Cl^−^], **e**: [Na^+^]) after 22 days of exposure to light treatments. Solid points and error bars display the means ± 95% confidence intervals from GLMMs, while faded points present the individual measurements after 24 h acute exposure to 34.5 ppt seawater. Open points display the means ± 95% confidence intervals from control samples collected in freshwater. High light intensity groups are presented in orange, while the low light intensity groups are presented in green. *Denotes a significant difference between the high and low light intensity treatments after the saltwater exposure.

Probability of maturation increased with increasing night light % in both the High and Low light intensity groups, but for the 1% and 10% groups was lower in the Low intensity treatment than High (Fig. 3, Table 2). The complete lack of maturation in the Low1 group, while some fish were maturating in the Low10 group, suggests a fixed in vivo minimum light detection threshold between 0.01 and 0.1 µmol m^−2^ s^−1^. Comparison of the GSI response ratios revealed that GSI in the Low1 group (mean 0.04, range 0.01–0.09, N = 174) was more similar to that of the High LD (0%) (mean 0.04, range < 0.001–0.11, N = 176) and Low LD (0%) (mean 0.05, range 0.02–0.10, N = 179) control groups than the High1 group (mean 0.42, range 0.01–7.4, N = 174). The same pattern was evident in the smoltification data. After 22 days of exposure to the experimental light regimes in freshwater, there were no significant differences in plasma concentrations of lactate, glucose, Ca^++^, K^+^, Cl^−^, nor osmolality between groups, though [cortisol] was significantly lower in both LD groups (0) and the Low1 group than in all other groups (Fig. 4). After the 24 h seawater challenge, there remained no significant differences between the High and Low regimes in plasma concentrations of lactate, glucose, Ca^++^ or K^+^. Plasma cortisol concentration increased in all groups during the seawater challenge but was significantly lower in both LD (0) groups and the Low1 group than all other groups (Fig. 5). Plasma osmolality, [Cl^−^] and [Na^+^] also increased in all groups during the seawater challenge and were significantly higher in both LD groups and the Low1 group than all other groups (Fig. 5c–e). Further, the patterns were similar for change in condition factor and SGR. Condition factor was unchanged in both LD groups and the Low1 group, whereas it decreased in all other groups (Fig. 5a). Similarly, SGR was higher in both LD groups and the Low1 group than the rest (Fig. 5b). Detailed numerical results from the significance testing can be found in supplementary table S1.

**Table 2:** Results of the generalized linear mixed model for probability of maturation.

Maturation GLMM	Estimate	SE	z value
Intercept	− 23.217	9140	− 0.003
High 1	21.530	9140	0.002
High 10	22.032	9140	0.002
High 100 (LL)	22.962	9140	0.003
Low 0 (LD)	− 0.472	14,661	0.000
Low 1	− 0.499	14,571	0.000
Low 10	20.567	9140	0.002
Low 100 (LL)	23.146	9140	0.003
Initial weight	0.545	0.11	5.053
Family—B	0.402	0.33	1.232
Family—C	− 0.241	0.27	− 0.880
Family—D	− 1.956	0.37	− 5.273

Estimate, standard error (SE) and z value of the explanatory variables.

### Photoperiod perception

However, if photoperiod interpretation was purely fixed based on a lower detection threshold the frequency of maturation and smoltification would be expected to increase with exposure to higher light intensities, at least up until a maximum plateau is reached. Instead, there was no difference in frequency of maturation between the High1 group (69L: 0.6D µmol m^−2^ s^−1^) and the Low10 group (1L: 0.1D µmol m^−2^ s^−1^), despite both the day and night light intensities being brighter in the High1 group. Similarly, more fish also matured in the LowLL group than the High10 group, despite being exposed to lower light intensities (Fig. 2). The largest difference in probability of maturation between the High and Low light intensity treatments was in the 10%-night groups, where only 6% of fish were maturing in the Low10 group while 20% were maturing in the High10 group. Similarly, although no fish were maturing in the Low1 group for any weight or family, on average 11% of fish were maturing in the High1 group.

### Impact of size and family on sexual maturation

The data also show that photoperiod interpretation varies between even closely related families. At the start of the experiment, there were no differences in weight (101 ± 19 g; *df* = 7, F = 0.38, *P* = 0.91) or length (20.2 ± 1.2 cm; *df* = 7, F = 0.67, *P* = 0.69) between light treatment groups. However, there were significant differences in initial weights (mean ± SD) between families (*df* = 3, F = 204.1, *P* =  < 0.0001), with families A (110 ± 11 g) and B (120 ± 13 g) being larger than families C (100 ± 19 g) and D (91 ± 14 g). Predicted probability of maturation increased with larger size in all families and light regimes where maturation occurred, but even so fish in family D were less likely to mature than fish in families A, B or C at any given size (Fig. 3).

## Discussion

Although light intensity varies on daily, seasonal and latitudinal scales, few studies have examined how recent light exposure history affects photoperiod interpretation in animals. These data experimentally demonstrate that Atlantic salmon have adaptive photoperiod interpretation responsive to light intensity variations and modification of the timing of key life history events as a result of adaptive photoperiod interpretation.

Salmonids possess an endogenous circannual clock which under natural conditions is continuously entrained by the seasonally changing daylength; daylength, in turn, is directly sensed via light exposure^24–30^. As a result, in salmon exposure to long days early in the year leads to phase advances, while long days after the summer solstice cause phase delays^3,27,28,31^. Since smoltification and sexual maturation can occur in parallel in Atlantic salmon^20^, by exposing parr to short followed by long photoperiods while held at a relatively warm temperature (16 °C) we initiated a phase advance expected to trigger both smoltification and sexual maturation^16,32^.

Fish in the LL, 10% and High1 groups exhibited slower growth and had lower condition factor than fish in the LD (0) and Low1 groups, suggesting they were devoting energetic resources to development other than growth. Similarly, the significantly higher plasma ion concentrations in the LD and Low1 groups compared to fish in the High1, 10 and LL groups after seawater challenge demonstrate an inability to maintain osmotic balance and lack of smoltification (Figs. 2 and 3). Thus, in both the smoltification and sexual maturation data, we detected a fixed in vivo minimum light detection threshold between 0.01 and 0.1 µmol m^−2^ s^−1^, in accordance with previous studies which pinpoint the threshold between 0.05 and 0.07 µmol m^−2^ s^−1^^11,17^.

However, if photoperiod interpretation were simply fixed above the minimum detection threshold then the frequency of sexual maturation would not differ between groups which experienced 24 h light exposure exceeding threshold. Instead, despite both the day and night light intensities being brighter in the High1 group, frequency of maturation did not differ with the Low10 group. Similarly, more fish also matured in the LowLL group than the High10 group (Fig. 2). Thus, these results confirm previous speculation that photoperiod interpretation in Atlantic salmon is flexible and adapts to variations in light intensity exposure above the minimum detection threshold^33,34^.

Given the varied conditions experienced by individual salmon throughout their lifecycle, including long distance migrations in both river and marine habitats, it is unsurprising that photoperiod interpretation in such a species would adapt based on recent exposure. It is also logically consistent that photoperiod interpretation would vary between populations for a species with a range distribution in excess of 30° (3000 km). The families included in this study all originate from strains which were lab bred for several generations, and even still distinct differences were apparent between families. Given the fidelity of salmon to their natal rivers^35^, it is possible that even larger differences in photoperiod interpretation exist between latitudinally distant wild populations.

In the simplest conceptualization of adaptive photoperiod interpretation, as long as the light intensity exceeds the minimum detection threshold, the intensity below which conditions would be interpreted as ‘darkness’ would vary directly with the light intensity in the previously experienced ‘day’. For example, in this experiment, frequency of maturation would be the same in the High and Low intensity treatments when the night: day intensity ratios were the same. However, despite both the High and Low intensity treatments exceeding the minimum detection threshold in the 10% treatments, more than three-times as many fish were maturing in the High10 group as in the paired Low10 group. Thus, further work is required to determine precisely how perception is adjusted in response to recent exposure.

Optimal timing of migration and reproduction are critical to both individual survival and species persistence^36^, and these results demonstrate the dramatic influence that even subtle changes in light exposure can have on phenological timing. As climate change alters the fitness landscape for species around the globe, conflicting environmental cues are expected to be one of the major threats facing temperate animals^37^. In salmon it is primarily salinity which stimulates the onset of spermatogenesis, while photoperiod modulates completion^32^. As a result, mismatch between salinity and daylength cues could rapidly lead to suboptimal distribution of energetic resources or ill timed reproductive maturity. At particular risk are species with already declining populations where the potential for rapid evolution is constrained due to small numbers^38,39^. Further work to elucidate the mechanisms of circannual timekeeping in teleosts, and exploration of the interactive effects of temperature and light intensity on phenological timing, are critical to understanding how species will respond to their changing environment.

## Supplementary Information

Supplementary Table S1.

## Data Availability

The datasets used and/or analysed during the current study are available from the corresponding author on reasonable request.
