# Supplementary material for: Adaptive photoperiod interpretation modulates phenological timing in Atlantic salmon
Source: Sci Rep. 2023 Feb 14;13:2618. doi: 10.1038/s41598-023-27583-7 (PMC9929253; doi:10.1038/s41598-023-27583-7)
Supplement: Supplementary file 1 — Supplementary Table S1. [file 41598_2023_27583_MOESM1_ESM.pdf]

Adaptive photoperiod interpretation modulates phenological timing in Atlantic salmon, by:Tina Oldham, Frode Oppedal, Per Gunnar Fjelldal and Tom Johnny Hansen

Supplementary Table S1 - contrasts of estimated marginal means with Tukey-adjusted P-value correction for multiple comparisons of plasma parameters in freshwater (0 ppt) and seawater (34.5 ppt).

[Lactate]						
Salinity (ppt)	contrast	ratio	SE	df	t.ratio	p.value
0	HighLD / LowLD	0.96	0.08	354	-0.52	1.000
0	High1 / Low1	1.10	0.10	354	1.11	0.999
0	High10 / Low10	1.02	0.07	354	0.30	1.000
0	HighLL / LowLL	0.99	0.09	354	-0.13	1.000
34.5	HighLD / High1	0.82	0.07	354	-2.28	0.641
34.5	HighLD / High10	0.84	0.07	354	-2.02	0.812
34.5	HighLD / HighLL	0.74	0.06	354	-3.47	0.048
34.5	HighLD / LowLD	0.96	0.08	354	-0.52	1.000
34.5	HighLD / Low1	0.90	0.08	354	-1.17	0.999
34.5	HighLD / Low10	0.86	0.07	354	-1.79	0.918
34.5	HighLD / LowLL	0.73	0.05	354	-4.83	0.000
34.5	High1 / High10	1.02	0.09	354	0.25	1.000
34.5	High1 / HighLL	0.90	0.08	354	-1.19	0.998
34.5	High1 / LowLD	1.17	0.07	354	2.40	0.548
34.5	High1 / Low1	1.10	0.10	354	1.11	0.999
34.5	High1 / Low10	1.04	0.09	354	0.47	1.000
34.5	High1 / LowLL	0.89	0.08	354	-1.32	0.995
34.5	High10 / HighLL	0.88	0.08	354	-1.43	0.988
34.5	High10 / LowLD	1.14	0.10	354	1.51	0.980
34.5	High10 / Low1	1.08	0.09	354	0.86	1.000
34.5	High10 / Low10	1.02	0.07	354	0.30	1.000
34.5	High10 / LowLL	0.87	0.08	354	-1.56	0.974
34.5	HighLL / LowLD	1.29	0.11	354	2.96	0.190
34.5	HighLL / Low1	1.22	0.08	354	3.11	0.132
34.5	HighLL / Low10	1.15	0.10	354	1.65	0.958
34.5	HighLL / LowLL	0.99	0.09	354	-0.13	1.000
34.5	LowLD / Low1	0.95	0.08	354	-0.65	1.000
34.5	LowLD / Low10	0.89	0.08	354	-1.29	0.996
34.5	LowLD / LowLL	0.77	0.07	354	-3.08	0.141
34.5	Low1 / Low10	0.95	0.08	354	-0.63	1.000
34.5	Low1 / LowLL	0.81	0.07	354	-2.42	0.531
34.5	Low10 / LowLL	0.86	0.07	354	-1.78	0.923

[Cl^-]						
Salinity (ppt)	contrast	ratio	SE	df	t.ratio	p.value
0	HighLD / LowLD	1.04	0.03	354	1.36	0.993
0	High1 / Low1	1.07	0.03	354	2.31	0.614
0	High10 / Low10	1.07	0.03	354	2.51	0.467
0	HighLL / LowLL	1.00	0.03	354	0.06	1.000
34.5	HighLD / High1	0.98	0.03	354	-0.55	1.000
34.5	HighLD / High10	0.99	0.03	354	-0.34	1.000
34.5	HighLD / HighLL	0.97	0.03	354	-0.88	1.000
34.5	HighLD / LowLD	1.04	0.03	354	1.36	0.993
34.5	HighLD / Low1	1.06	0.03	354	1.77	0.926
34.5	HighLD / Low10	1.06	0.03	354	1.84	0.899
34.5	HighLD / LowLL	0.98	0.03	354	-0.94	1.000
34.5	High1 / High10	1.01	0.03	354	0.20	1.000
34.5	High1 / HighLL	0.99	0.03	354	-0.33	1.000
34.5	High1 / LowLD	1.06	0.03	354	2.21	0.689
34.5	High1 / Low1	1.07	0.03	354	2.31	0.614
34.5	High1 / Low10	1.08	0.03	354	2.38	0.560
34.5	High1 / LowLL	0.99	0.03	354	-0.27	1.000
34.5	High10 / HighLL	0.98	0.03	354	-0.53	1.000
34.5	High10 / LowLD	1.05	0.03	354	1.70	0.946
34.5	High10 / Low1	1.07	0.03	354	2.11	0.761
34.5	High10 / Low10	1.07	0.03	354	2.51	0.467
34.5	High10 / LowLL	0.99	0.03	354	-0.47	1.000
34.5	HighLL / LowLD	1.07	0.03	354	2.24	0.667
34.5	HighLL / Low1	1.08	0.03	354	3.07	0.147
34.5	HighLL / Low10	1.09	0.03	354	2.72	0.325
34.5	HighLL / LowLL	1.00	0.03	354	0.06	1.000
34.5	LowLD / Low1	1.01	0.03	354	0.42	1.000
34.5	LowLD / Low10	1.02	0.03	354	0.50	1.000
34.5	LowLD / LowLL	0.94	0.03	354	-2.17	0.719
34.5	Low1 / Low10	1.00	0.03	354	0.09	1.000
34.5	Low1 / LowLL	0.92	0.03	354	-2.57	0.421
34.5	Low10 / LowLL	0.92	0.03	354	-2.64	0.372

[Glucose]						
Salinity (ppt)	contrast	ratio	SE	df	t.ratio	p.value
0	HighLD / LowLD	1.04	0.03	354	1.36	0.993
0	High1 / Low1	1.07	0.03	354	2.31	0.614
0	High10 / Low10	1.07	0.03	354	2.51	0.467
0	HighLL / LowLL	1.00	0.03	354	0.06	1.000
34.5	HighLD / High1	0.98	0.03	354	-0.55	1.000
34.5	HighLD / High10	0.99	0.03	354	-0.34	1.000
34.5	HighLD / HighLL	0.97	0.03	354	-0.88	1.000
34.5	HighLD / LowLD	1.04	0.03	354	1.36	0.993
34.5	HighLD / Low1	1.06	0.03	354	1.77	0.926
34.5	HighLD / Low10	1.06	0.03	354	1.84	0.899
34.5	HighLD / LowLL	0.98	0.03	354	-0.94	1.000
34.5	High1 / High10	1.01	0.03	354	0.20	1.000
34.5	High1 / HighLL	0.99	0.03	354	-0.33	1.000
34.5	High1 / LowLD	1.06	0.03	354	2.21	0.689
34.5	High1 / Low1	1.07	0.03	354	2.31	0.614
34.5	High1 / Low10	1.08	0.03	354	2.38	0.560
34.5	High1 / LowLL	0.99	0.03	354	-0.27	1.000
34.5	High10 / HighLL	0.98	0.03	354	-0.53	1.000
34.5	High10 / LowLD	1.05	0.03	354	1.70	0.946
34.5	High10 / Low1	1.07	0.03	354	2.11	0.761
34.5	High10 / Low10	1.07	0.03	354	2.51	0.467
34.5	High10 / LowLL	0.99	0.03	354	-0.47	1.000
34.5	HighLL / LowLD	1.07	0.03	354	2.24	0.667
34.5	HighLL / Low1	1.08	0.03	354	3.07	0.147
34.5	HighLL / Low10	1.09	0.03	354	2.72	0.325
34.5	HighLL / LowLL	1.00	0.03	354	0.06	1.000
34.5	LowLD / Low1	1.01	0.03	354	0.42	1.000
34.5	LowLD / Low10	1.02	0.03	354	0.50	1.000
34.5	LowLD / LowLL	0.94	0.03	354	-2.17	0.719
34.5	Low1 / Low10	1.00	0.03	354	0.09	1.000
34.5	Low1 / LowLL	0.92	0.03	354	-2.57	0.421
34.5	Low10 / LowLL	0.92	0.03	354	-2.64	0.372

[Na^+]						
Salinity (ppt)	contrast	ratio	SE	df	t.ratio	p.value
0	HighLD / LowLD	1.00	0.01	354	0.43	1.000
0	High1 / Low1	0.98	0.01	354	-4.18	0.004
0	High10 / Low10	1.00	0.01	354	0.18	1.000
0	HighLL / LowLL	1.00	0.01	354	0.83	1.000
34.5	HighLD / High1	1.06	0.01	354	9.14	0.000
34.5	HighLD / High10	1.06	0.01	354	9.90	0.000
34.5	HighLD / HighLL	1.05	0.01	354	7.70	0.000
34.5	HighLD / LowLD	1.00	0.01	354	0.43	1.000
34.5	HighLD / Low1	1.03	0.01	354	4.96	0.000
34.5	HighLD / Low10	1.06	0.01	354	10.03	0.000
34.5	HighLD / LowLL	1.05	0.01	354	8.49	0.000
34.5	High1 / High10	1.00	0.01	354	0.81	1.000
34.5	High1 / HighLL	0.99	0.01	354	-1.44	0.988
34.5	High1 / LowLD	0.95	0.01	354	-8.76	0.000
34.5	High1 / Low1	0.98	0.01	354	-4.18	0.004
34.5	High1 / Low10	1.01	0.01	354	0.99	1.000
34.5	High1 / LowLL	1.00	0.01	354	-0.60	1.000
34.5	High10 / HighLL	0.99	0.01	354	-2.24	0.668
34.5	High10 / LowLD	0.94	0.01	354	-9.52	0.000
34.5	High10 / Low1	0.97	0.01	354	-4.97	0.000
34.5	High10 / Low10	1.00	0.01	354	0.18	1.000
34.5	High10 / LowLL	0.99	0.01	354	-1.41	0.990
34.5	HighLL / LowLD	0.96	0.01	354	-7.31	0.000
34.5	HighLL / Low1	0.98	0.01	354	-2.74	0.308
34.5	HighLL / Low10	1.01	0.01	354	2.41	0.540
34.5	HighLL / LowLL	1.00	0.01	354	0.83	1.000
34.5	LowLD / Low1	1.03	0.01	354	4.55	0.001
34.5	LowLD / Low10	1.06	0.01	354	9.65	0.000
34.5	LowLD / LowLL	1.05	0.01	354	8.10	0.000
34.5	Low1 / Low10	1.03	0.01	354	5.12	0.000
34.5	Low1 / LowLL	1.02	0.01	354	3.55	0.036
34.5	Low10 / LowLL	0.99	0.01	354	-1.58	0.971

$[Ca^{++}]$						
Salinity (ppt)	contrast	ratio	SE	df	t.ratio	p.value
0	HighLD / LowLD	1.01	0.03	354	0.17	1.000
0	High1 / Low1	0.96	0.03	354	-1.37	0.992
0	High10 / Low10	1.04	0.03	354	1.18	0.998
0	HighLL / LowLL	0.99	0.03	354	-0.17	1.000
34.5	HighLD / High1	1.06	0.04	354	1.64	0.960
34.5	HighLD / High10	1.08	0.04	354	2.17	0.718
34.5	HighLD / HighLL	1.04	0.03	354	1.32	0.995
34.5	HighLD / LowLD	1.01	0.03	354	0.17	1.000
34.5	HighLD / Low1	1.01	0.03	354	0.27	1.000
34.5	HighLD / Low10	1.12	0.04	354	3.28	0.082
34.5	HighLD / LowLL	1.04	0.03	354	1.21	0.998
34.5	High1 / High10	1.02	0.03	354	0.54	1.000
34.5	High1 / HighLL	0.99	0.03	354	-0.32	1.000
34.5	High1 / LowLD	0.95	0.03	354	-1.56	0.973
34.5	High1 / Low1	0.96	0.03	354	-1.37	0.992
34.5	High1 / Low10	1.06	0.04	354	1.66	0.955
34.5	High1 / LowLL	0.98	0.03	354	-0.49	1.000
34.5	High10 / HighLL	0.97	0.03	354	-0.86	1.000
34.5	High10 / LowLD	0.94	0.03	354	-2.01	0.818
34.5	High10 / Low1	0.94	0.03	354	-1.90	0.873
34.5	High10 / Low10	1.04	0.03	354	1.18	0.998
34.5	High10 / LowLL	0.97	0.03	354	-1.02	1.000
34.5	HighLL / LowLD	0.96	0.03	354	-1.16	0.999
34.5	HighLL / Low1	0.97	0.03	354	-1.11	0.999
34.5	HighLL / Low10	1.07	0.04	354	1.98	0.836
34.5	HighLL / LowLL	0.99	0.03	354	-0.17	1.000
34.5	LowLD / Low1	1.00	0.03	354	0.10	1.000
34.5	LowLD / Low10	1.11	0.04	354	3.13	0.125
34.5	LowLD / LowLL	1.03	0.03	354	0.98	1.000
34.5	Low1 / Low10	1.11	0.04	354	3.02	0.166
34.5	Low1 / LowLL	1.03	0.03	354	0.88	1.000
34.5	Low10 / LowLL	0.93	0.03	354	-2.14	0.741

Osmolality						
Salinity (ppt)	contrast	ratio	SE	df	t.ratio	p.value
0	HighLD / LowLD	1.01	0.01	354	0.99	1.000
0	High1 / Low1	0.98	0.01	354	-3.79	0.016
0	High10 / Low10	1.00	0.01	354	0.78	1.000
0	HighLL / LowLL	1.00	0.01	354	0.73	1.000
34.5	HighLD / High1	1.05	0.01	354	7.28	0.000
34.5	HighLD / High10	1.05	0.01	354	7.54	0.000
34.5	HighLD / HighLL	1.04	0.01	354	5.88	0.000
34.5	HighLD / LowLD	1.01	0.01	354	0.99	1.000
34.5	HighLD / Low1	1.02	0.01	354	3.49	0.044
34.5	HighLD / Low10	1.05	0.01	354	8.29	0.000
34.5	HighLD / LowLL	1.04	0.01	354	6.58	0.000
34.5	High1 / High10	1.00	0.01	354	0.30	1.000
34.5	High1 / HighLL	0.99	0.01	354	-1.40	0.991
34.5	High1 / LowLD	0.96	0.01	354	-6.33	0.000
34.5	High1 / Low1	0.98	0.01	354	-3.79	0.016
34.5	High1 / Low10	1.01	0.01	354	1.09	0.999
34.5	High1 / LowLL	1.00	0.01	354	-0.66	1.000
34.5	High10 / HighLL	0.99	0.01	354	-1.70	0.947
34.5	High10 / LowLD	0.96	0.01	354	-6.60	0.000
34.5	High10 / Low1	0.97	0.01	354	-4.07	0.006
34.5	High10 / Low10	1.00	0.01	354	0.78	1.000
34.5	High10 / LowLL	0.99	0.01	354	-0.96	1.000
34.5	HighLL / LowLD	0.97	0.01	354	-4.92	0.000
34.5	HighLL / Low1	0.99	0.01	354	-2.39	0.558
34.5	HighLL / Low10	1.02	0.01	354	2.48	0.492
34.5	HighLL / LowLL	1.00	0.01	354	0.73	1.000
34.5	LowLD / Low1	1.02	0.01	354	2.52	0.456
34.5	LowLD / Low10	1.05	0.01	354	7.36	0.000
34.5	LowLD / LowLL	1.04	0.01	354	5.63	0.000
34.5	Low1 / Low10	1.03	0.01	354	4.84	0.000
34.5	Low1 / LowLL	1.02	0.01	354	3.11	0.133
34.5	Low10 / LowLL	0.99	0.01	354	-1.74	0.935

[K^+]						
Salinity (ppt)	contrast	ratio	SE	df	t.ratio	p.value
0	HighLD / LowLD	1.01	0.05	354	0.24	1.000
0	High1 / Low1	0.98	0.05	354	-0.47	1.000
0	High10 / Low10	1.00	0.04	354	0.04	1.000
0	HighLL / LowLL	1.03	0.05	354	0.61	1.000
34.5	HighLD / High1	1.04	0.05	354	0.87	1.000
34.5	HighLD / High10	1.04	0.05	354	0.86	1.000
34.5	HighLD / HighLL	1.00	0.05	354	-0.03	1.000
34.5	HighLD / LowLD	1.01	0.05	354	0.24	1.000
34.5	HighLD / Low1	1.02	0.05	354	0.40	1.000
34.5	HighLD / Low10	1.04	0.05	354	0.89	1.000
34.5	HighLD / LowLL	1.03	0.04	354	0.75	1.000
34.5	High1 / High10	1.00	0.05	354	-0.01	1.000
34.5	High1 / HighLL	0.96	0.05	354	-0.90	1.000
34.5	High1 / LowLD	0.97	0.03	354	-0.84	1.000
34.5	High1 / Low1	0.98	0.05	354	-0.47	1.000
34.5	High1 / Low10	1.00	0.05	354	0.02	1.000
34.5	High1 / LowLL	0.99	0.05	354	-0.29	1.000
34.5	High10 / HighLL	0.96	0.05	354	-0.89	1.000
34.5	High10 / LowLD	0.97	0.05	354	-0.63	1.000
34.5	High10 / Low1	0.98	0.05	354	-0.46	1.000
34.5	High10 / Low10	1.00	0.04	354	0.04	1.000
34.5	High10 / LowLL	0.99	0.05	354	-0.28	1.000
34.5	HighLL / LowLD	1.01	0.05	354	0.27	1.000
34.5	HighLL / Low1	1.02	0.04	354	0.56	1.000
34.5	HighLL / Low10	1.04	0.05	354	0.91	1.000
34.5	HighLL / LowLL	1.03	0.05	354	0.61	1.000
34.5	LowLD / Low1	1.01	0.05	354	0.16	1.000
34.5	LowLD / Low10	1.03	0.05	354	0.65	1.000
34.5	LowLD / LowLL	1.02	0.05	354	0.34	1.000
34.5	Low1 / Low10	1.02	0.05	354	0.49	1.000
34.5	Low1 / LowLL	1.01	0.05	354	0.18	1.000
34.5	Low10 / LowLL	0.99	0.05	354	-0.31	1.000

[Cortisol]						
Salinity (ppt)	contrast	ratio	SE	df	t.ratio	p.value
0	HighLD / LowLD	1.16	0.13	354	1.36	0.993
0	High1 / Low1	1.80	0.20	354	5.17	0.000
0	High10 / Low10	1.16	0.13	354	1.39	0.992
0	HighLL / LowLL	1.19	0.13	354	1.53	0.979
34.5	HighLD / High1	0.52	0.06	354	-5.71	0.000
34.5	HighLD / High10	0.53	0.06	354	-5.51	0.000
34.5	HighLD / HighLL	0.49	0.06	354	-6.34	0.000
34.5	HighLD / LowLD	1.16	0.13	354	1.36	0.993
34.5	HighLD / Low1	0.94	0.11	354	-0.53	1.000
34.5	HighLD / Low10	0.62	0.07	354	-4.18	0.004
34.5	HighLD / LowLL	0.58	0.06	354	-5.09	0.000
34.5	High1 / High10	1.02	0.12	354	0.17	1.000
34.5	High1 / HighLL	0.93	0.11	354	-0.64	1.000
34.5	High1 / LowLD	2.22	0.24	354	7.51	0.000
34.5	High1 / Low1	1.80	0.20	354	5.17	0.000
34.5	High1 / Low10	1.18	0.14	354	1.48	0.984
34.5	High1 / LowLL	1.11	0.13	354	0.89	1.000
34.5	High10 / HighLL	0.91	0.10	354	-0.81	1.000
34.5	High10 / LowLD	2.18	0.25	354	6.88	0.000
34.5	High10 / Low1	1.76	0.20	354	4.99	0.000
34.5	High10 / Low10	1.16	0.13	354	1.39	0.992
34.5	High10 / LowLL	1.08	0.12	354	0.71	1.000
34.5	HighLL / LowLD	2.39	0.27	354	7.71	0.000
34.5	HighLL / Low1	1.93	0.21	354	6.17	0.000
34.5	HighLL / Low10	1.27	0.15	354	2.12	0.753
34.5	HighLL / LowLL	1.19	0.13	354	1.53	0.979
34.5	LowLD / Low1	0.81	0.09	354	-1.89	0.878
34.5	LowLD / Low10	0.53	0.06	354	-5.54	0.000
34.5	LowLD / LowLL	0.50	0.06	354	-6.15	0.000
34.5	Low1 / Low10	0.66	0.08	354	-3.65	0.026
34.5	Low1 / LowLL	0.62	0.07	354	-4.27	0.003
34.5	Low10 / LowLL	0.93	0.11	354	-0.60	1.000

Condition Factor						
Salinity (ppt)	contrast	estimat	SE	df	t.ratio	p.value
0	HighLD / LowLD	-0.01	0.02	354	-0.49	1.000
0	High1 / Low1	-0.17	0.02	354	-11.48	0.000
0	High10 / Low10	0.01	0.02	354	0.72	1.000
0	HighLL / LowLL	-0.02	0.02	354	-1.25	0.997
34.5	HighLD / High1	0.13	0.02	354	8.78	0.000
34.5	HighLD / High10	0.11	0.02	354	7.42	0.000
34.5	HighLD / HighLL	0.12	0.02	354	8.14	0.000
34.5	HighLD / LowLD	-0.01	0.02	354	-0.49	1.000
34.5	HighLD / Low1	-0.04	0.02	354	-2.70	0.332
34.5	HighLD / Low10	0.12	0.02	354	8.10	0.000
34.5	HighLD / LowLL	0.11	0.02	354	6.85	0.000
34.5	High1 / High10	-0.02	0.02	354	-1.31	0.995
34.5	High1 / HighLL	-0.01	0.02	354	-0.63	1.000
34.5	High1 / LowLD	-0.14	0.02	354	-9.31	0.000
34.5	High1 / Low1	-0.17	0.02	354	-11.48	0.000
34.5	High1 / Low10	-0.01	0.02	354	-0.58	1.000
34.5	High1 / LowLL	-0.03	0.02	354	-1.88	0.885
34.5	High10 / HighLL	0.01	0.02	354	0.68	1.000
34.5	High10 / LowLD	-0.12	0.02	354	-7.95	0.000
34.5	High10 / Low1	-0.15	0.02	354	-10.11	0.000
34.5	High10 / Low10	0.01	0.02	354	0.72	1.000
34.5	High10 / LowLL	-0.01	0.02	354	-0.57	1.000
34.5	HighLL / LowLD	-0.13	0.02	354	-8.68	0.000
34.5	HighLL / Low1	-0.17	0.02	354	-10.85	0.000
34.5	HighLL / Low10	0.00	0.02	354	0.05	1.000
34.5	HighLL / LowLL	-0.02	0.02	354	-1.25	0.997
34.5	LowLD / Low1	-0.03	0.02	354	-2.23	0.675
34.5	LowLD / Low10	0.13	0.02	354	8.63	0.000
34.5	LowLD / LowLL	0.11	0.02	354	7.37	0.000
34.5	Low1 / Low10	0.17	0.02	354	10.77	0.000
34.5	Low1 / LowLL	0.15	0.02	354	9.54	0.000
34.5	Low10 / LowLL	-0.02	0.02	354	-1.28	0.996

SGR						
Salinity (ppt)	contrast	estimat	SE	df	t.ratio	p.value
0	HighLD / LowLD	-0.05	0.10	354	-0.45	1.000
0	High1 / Low1	-1.08	0.10	354	-10.32	0.000
0	High10 / Low10	0.15	0.11	354	1.44	0.988
0	HighLL / LowLL	-0.15	0.11	354	-1.44	0.988
34.5	HighLD / High1	0.53	0.10	354	5.02	0.000
34.5	HighLD / High10	0.32	0.11	354	3.04	0.160
34.5	HighLD / HighLL	0.40	0.10	354	3.87	0.012
34.5	HighLD / LowLD	-0.05	0.10	354	-0.45	1.000
34.5	HighLD / Low1	-0.55	0.10	354	-5.29	0.000
34.5	HighLD / Low10	0.47	0.11	354	4.46	0.001
34.5	HighLD / LowLL	0.25	0.11	354	2.40	0.547
34.5	High1 / High10	-0.21	0.11	354	-1.96	0.846
34.5	High1 / HighLL	-0.12	0.10	354	-1.16	0.999
34.5	High1 / LowLD	-0.57	0.10	354	-5.50	0.000
34.5	High1 / Low1	-1.08	0.10	354	-10.32	0.000
34.5	High1 / Low10	-0.05	0.11	354	-0.50	1.000
34.5	High1 / LowLL	-0.27	0.11	354	-2.59	0.408
34.5	High10 / HighLL	0.09	0.11	354	0.81	1.000
34.5	High10 / LowLD	-0.37	0.10	354	-3.50	0.043
34.5	High10 / Low1	-0.87	0.11	354	-8.30	0.000
34.5	High10 / Low10	0.15	0.11	354	1.44	0.988
34.5	High10 / LowLL	-0.07	0.11	354	-0.63	1.000
34.5	HighLL / LowLD	-0.45	0.10	354	-4.33	0.002
34.5	HighLL / Low1	-0.96	0.10	354	-9.16	0.000
34.5	HighLL / Low10	0.07	0.11	354	0.64	1.000
34.5	HighLL / LowLL	-0.15	0.11	354	-1.44	0.988
34.5	LowLD / Low1	-0.51	0.10	354	-4.87	0.000
34.5	LowLD / Low10	0.52	0.11	354	4.93	0.000
34.5	LowLD / LowLL	0.30	0.10	354	2.86	0.241
34.5	Low1 / Low10	1.03	0.11	354	9.70	0.000
34.5	Low1 / LowLL	0.81	0.11	354	7.67	0.000
34.5	Low10 / LowLL	-0.22	0.11	354	-2.06	0.787

pH						
Salinity (ppt)	contrast	ratio	SE	df	t.ratio	p.value
0	HighLD / LowLD	1.00	0.00	354	-0.19	1.000
0	High1 / Low1	1.01	0.00	354	6.17	0.000
0	High10 / Low10	1.00	0.00	354	0.81	1.000
0	HighLL / LowLL	1.00	0.00	354	-0.36	1.000
34.5	HighLD / High1	0.99	0.00	354	-4.05	0.006
34.5	HighLD / High10	0.99	0.00	354	-3.29	0.080
34.5	HighLD / HighLL	0.99	0.00	354	-2.75	0.301
34.5	HighLD / LowLD	1.00	0.00	354	-0.19	1.000
34.5	HighLD / Low1	1.00	0.00	354	2.13	0.748
34.5	HighLD / Low10	1.00	0.00	354	-2.46	0.507
34.5	HighLD / LowLL	0.99	0.00	354	-3.10	0.136
34.5	High1 / High10	1.00	0.00	354	0.73	1.000
34.5	High1 / HighLL	1.00	0.00	354	1.29	0.996
34.5	High1 / LowLD	1.01	0.00	354	3.88	0.012
34.5	High1 / Low1	1.01	0.00	354	6.17	0.000
34.5	High1 / Low10	1.00	0.00	354	1.55	0.976
34.5	High1 / LowLL	1.00	0.00	354	0.92	1.000
34.5	High10 / HighLL	1.00	0.00	354	0.55	1.000
34.5	High10 / LowLD	1.01	0.00	354	3.12	0.128
34.5	High10 / Low1	1.01	0.00	354	5.40	0.000
34.5	High10 / Low10	1.00	0.00	354	0.81	1.000
34.5	High10 / LowLL	1.00	0.00	354	0.19	1.000
34.5	HighLL / LowLD	1.01	0.00	354	2.58	0.416
34.5	HighLL / Low1	1.01	0.00	354	4.88	0.000
34.5	HighLL / Low10	1.00	0.00	354	0.27	1.000
34.5	HighLL / LowLL	1.00	0.00	354	-0.36	1.000
34.5	LowLD / Low1	1.00	0.00	354	2.33	0.605
34.5	LowLD / Low10	1.00	0.00	354	-2.28	0.638
34.5	LowLD / LowLL	0.99	0.00	354	-2.93	0.207
34.5	Low1 / Low10	0.99	0.00	354	-4.56	0.001
34.5	Low1 / LowLL	0.99	0.00	354	-5.21	0.000
34.5	Low10 / LowLL	1.00	0.00	354	-0.62	1.000
